# Euglycemic Ketoacidosis in a Patient with Metastatic Non-Small-Cell Lung Adenocarcinoma and Concomitant Pulmonary Embolism

**DOI:** 10.1155/2020/8882299

**Published:** 2020-08-07

**Authors:** Robert Sean O'Neill, Lauren Tyack, Mary Freeman, Hussein Soudy Hussein

**Affiliations:** ^1^The Department of Medical Oncology, The Sutherland Hospital, Caringbah, NSW, Australia; ^2^The Department of Endocrinology, The Sutherland Hospital, Caringbah, NSW, Australia; ^3^The Department of Medical Oncology, St George Hospital, Kogarah, NSW, Australia

## Abstract

Euglycemic ketoacidosis is a recognised side effect secondary to sodium-glucose cotransporter 2 inhibitor use in the treatment of type 2 diabetes mellitus; however, there is scarce evidence to suggest whether preexisting comorbid conditions contribute to the development of this potentially life-threatening complication. We describe a case of euglycemic ketoacidosis in a patient with type 2 diabetes mellitus in the context of empagliflozin use after a recent diagnosis of metastatic lung adenocarcinoma. The diagnosis was complicated by a pulmonary embolism and hospital-acquired pneumonia, and was subsequently established after an anion-gap metabolic acidosis was identified on arterial blood gas and serum ketone measurement. The patient required admission to the intensive care unit for fluid resuscitation and regular intravenous insulin to ensure resolution of acidosis and maintenance of normoglycaemia. The patient was discharged to home for outpatient single-agent pembrolizumab for treatment of his lung adenocarcinoma. This article highlights the importance or awareness of oral hypoglycaemic medications and their side effects, along with providing further evidence for the potential contribution of malignancy to the development of euglycemic ketoacidosis in a patient with type 2 diabetes mellitus.

## 1. Introduction

Sodium-glucose cotransporter 2 inhibitors (SGLT2i) are a new class of oral diabetic medications that have gained favour in recent times due to the reported favourable renal and cardiovascular outcomes; however, the full extent of their side-effect profile is not completely understood. The triad of euglycemia, ketosis, and anion-gap metabolic acidosis defines euglycemic ketoacidosis, a rare, yet lethal complication of SGLT2i therapy with rates ranging depending on the dose administered [1]. Diabetic ketoacidosis is known to occur in the context of depleted circulating insulin, a situation which can be precipitated by both a starvation state and an acute medical illness, which subsequently results in a state of catabolism highlighted by lipolysis, hyperglucagonemia, fatty acid oxidation, and finally ketosis. In the case of SGLT2i use, glycosuria is stimulated resulting in decreased serum glucose levels, producing euglycemia [2].

Here, we present the case of a male with type 2 diabetes mellitus (T2DM) and non-small-cell lung cancer (NSCLC) who developed euglycemic ketoacidosis in the context of empagliflozin use, after presenting with symptomatic hypercalcaemia, and was subsequently diagnosed with a pulmonary embolism.

## 2. Case Presentation

A 71-year-old male was referred to a metropolitan hospital for management of symptomatic hypercalcaemia. This was in the context of a recent diagnosis of metastatic stage IV lung adenocarcinoma and PDL-1 expression 50%, with baseline positron emission tomography (PET) scan demonstrating a left lingular lesion, and mediastinal and upper abdominal glucose avid nodes. In addition to this, avid disease was noted in the right hilum and bilateral supraclavicular regions. He had a background of invasive urinary bladder carcinoma previously treated with methotrexate, vinblastine sulfate, adriamycin, and cisplatin (MVAC) chemotherapy, formation of a ureteric ileostomy, and T2DM. With regard to the patient's diagnosis of T2DM, this was previously managed with diet and he had been diagnosed as an outpatient with a HbA1c of 9.0%. This had been escalated to oral metformin 500 mg twice daily, and in addition to this, empagliflozin 10 mg once daily had also been initiated three months prior to presentation due to labile blood sugar levels.

On preliminary assessment, he reported constipation and issues with short-term memory but was otherwise clinically well. Clinical examination demonstrated decreased air entry over his left lower lobe on thoracic auscultation. He was of short stature, weighing 75.3 kg with a body mass index (BMI) of 28.4 kg/m^2^, and he reported nil changes in his ileostomy output preceding admission.

**Figure 1:**
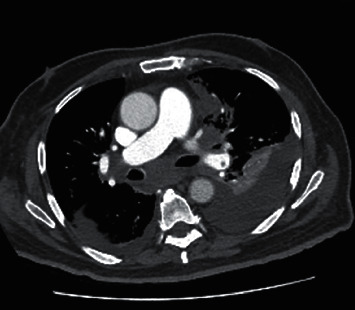
Transverse view of CTPA demonstrating bilateral pleural effusions with collapse consolidation and left pulmonary artery embolus.

His calcium level was 3.0 mmol/L on admission which was managed with a single dose of zoledronic acid and intravenous fluids with resolution on day 2 of admission. On day 2 of admission, the patient developed a fever to 38.3 degrees Celsius and was commenced on intravenous antibiotics due to suspicion of hospital acquired pneumonia (HAP). On day 4 of admission, the patient developed tachypnoea and was hypoxic to 88% on room air. Computed tomography pulmonary angiogram (CTPA) (Figure 1) demonstrated bilateral pulmonary emboli, a significant left-sided pleural effusion, and straightening of the ventricular septum indicating right heart strain which was confirmed on transthoracic echocardiogram with an estimated mean pulmonary artery pressure of 42 mmHg and a dilated right ventricle with impaired systolic function reported. The patient was subsequently commenced on therapeutic anticoagulation and was escalated to high flow nasal prongs for ventilatory support. Due to ongoing tachypnoea despite high flow oxygen supplementation, an arterial blood gas was taken which demonstrated an anion-gap metabolic acidosis (pH 7.343, pCO_2_ 25.7 mmHg, base excess −11.0 mmol/L, HCO_3_^−^ 13.6 mmol/L, and albumin-corrected anion gap 19.9 mmol/L) with bedside capillary glucose and ketones reported as 5.1 mmol/L and 7.4 mmol/L, respectively. Further investigation revealed that the patient's regular empagliflozin, a SGLT2i had been continued on admission.

The patient was commenced on an insulin and dextrose infusion and transferred to the intensive care unit (ICU) for further management. Repeat arterial blood gases were taken regularly throughout admission to the ICU, where he was maintained on a dextrose and insulin infusion until his metabolic acidosis resolved and normoglycemia, along with low-level serum ketonaemia maintained (Table 1). The patient's renal function remained normal throughout admission.

The patient underwent pleurocentesis on day 6 of admission for symptomatic relief with mild improvement in his oxygen requirement. Pleural fluid cytology was consistent with non-small-cell lung primary metastatic adenocarcinoma.

The patient was subsequently discharged from the ICU on day 7 of admission, upon which his serum ketones had normalised and his BSLs were controlled on a supplemental subcutaneous insulin regime. He had an ongoing oxygen requirement; however. his tachypnoea had resolved. He was transitioned from a heparin infusion to therapeutic clexane for management of his bilateral pulmonary emboli, and his chest X-ray on discharge from the ICU demonstrated a stable appearance in his bilateral pleural effusions after pleurocentesis.

The remainder of the patient's admission was unremarkable. He was discharged on day 15 of admission with palliative oxygen therapy for symptomatic relief and was referred for outpatient treatment with single-agent pembrolizumab. Empagliflozin was withheld on discharge, and due to adequate BSL control as an inpatient, insulin was ceased with a view to recommence outpatient low-dose metformin.

## 3. Discussion

The concept of euglycemic ketoacidosis was first proposed in 1973, with causes such as gestational diabetes, reduced calorie intake, excessive alcohol consumption, acute pancreatitis, and chronic hepatitis being reported in the literature [1, 3]. In addition to this, the introduction of SGLT2i in the management of T2DM has resulted in emerging cases of euglycemic ketoacidosis [4]. This is postulated to occur through decreasing fasting and postprandial glucose levels resulting in decreased insulin production and subsequently has been shown to promote hyperglucagonemia, along with the propagation of a negative fluid and sodium balance [4, 5]. In addition to this, subsequent glycosuria as a result of the action of SGLT2i results in normoglycaemia, presenting the biochemical phenotype that is euglycemic ketoacidosis. The mechanism of action of SGLT2i is independent of insulin and can be used at any stage of illness in T2DM, and in addition to promoting normoglycaemia, they have favourable effects in reducing body weight, blood pressure, reducing atherosclerotic cardiovascular events in patients with established cardiovascular disease, reducing heart failure hospitalisation rates and progression of renal disease [6, 7].

Euglycemic acidosis occurring in the context of SGLT2i use in patients with malignancy is scarce, with previous reports documenting its incidence in the case of colorectal cancer [8], pancreatic cancer [9], and lung cancer [10]. Although previous data have demonstrated that SGLT2i-associated euglycemic ketoacidosis is not associated with any particular demographic or comorbid population, perhaps further prospective studies are required to determine whether malignancy can further predispose to the development of euglycemic ketoacidosis and, additionally, whether certain malignancies are associated with an increased risk.

The presented case presents a fortunate and timely diagnosis of euglycemic ketoacidosis in a patient with complex health comorbidities. It is the authors' opinion that, although this was not the reason for his presentation, the development of a HAP, subsequent pulmonary embolism, and his existing thoracic malignancy predisposed for the development of euglycemic ketoacidosis.

In patients with complex comorbidities presenting with an acute illness, clinicians should address the need for continuing SGLT2i use during admission and should have a high index of suspicious for the development of euglycemic ketoacidosis with their use. Complications of their continued use in a stress state can result in severe consequences in the unwell patient.

## Figures and Tables

**Table 1:** Blood gas from the point of assessment on day 2 of admission demonstrating resolving metabolic acidosis.

	0	5 hours	12 hours	15 hours	20 hours^*∗*^
pH	7.343	7.348	7.391	7.365	7.384
pO_2_ (mmHg)	75.3	110.0	70.9	85.7	34.3
pCO_2_ (mmHg)	25.7	28.7	34.5	37.9	38.0
HCO_3_^−^ (mmol/L)	13.6	15.4	20.5	21.1	22.2
Base excess (mmol/L)	−11.0	−9.1	−3.6	−3.4	−2.1
BGL (mmol/L)	4.7	9.7	6.8	7.1	7.0
Blood ketone level (mmol/L)	7.4	4.7	0.1	1.1	0.3

^*∗*^Venous blood gas.

## Data Availability

No data were used to support the findings of this study.
